# Hallucinogenic Persisting Perception Disorder: A Case Series and Review of the Literature

**DOI:** 10.3389/fneur.2022.878609

**Published:** 2022-05-06

**Authors:** Hannah Ford, Clare L. Fraser, Emma Solly, Meaghan Clough, Joanne Fielding, Owen White, Anneke Van Der Walt

**Affiliations:** ^1^Department of Neurology, Alfred Health, Melbourne, VIC, Australia; ^2^Faculty of Health and Medicine, Save Sight Institute, The University of Sydney, Sydney, NSW, Australia; ^3^Department of Neuroscience, Central Clinical School, Monash University, Melbourne, VIC, Australia; ^4^Department of Neuro-Ophthalmology, Royal Victorian Eye and Ear Hospital, Melbourne, VIC, Australia

**Keywords:** neuro-ophthalmology, hallucinogenic persisting perception disorder, optical coherence tomography, neuropsychiatry, case series

## Abstract

**Background:**

Hallucinogen persisting perception disorder (HPPD) is characterized by the re-emergence of perceptual symptoms experienced during acute hallucinogen intoxication following drug cessation. The underlying pathophysiology is poorly understood. We report the clinical characteristics and investigation findings of a series of HPPD cases with a literature review of previous case reports. We draw parallels between the features of HPPD and Visual Snow Syndrome (VSS).

**Methods:**

Retrospective case series of 13 patients referred from neuro-ophthalmologists. Literature review with 24 HPPD case reports were identified through database search using the terms “hallucinogenic persisting perception disorder” OR “hallucinogen persisting perception disorder.”

**Results:**

Lysergic acid diethylamide (LSD), 3,4-Methyl enedioxy methamphetamine (MDMA) and cannabinoid use was common. Cannabinoids and MDMA were mostly used in association with classical hallucinogens. The most frequent symptoms in our patients were visual snow, floaters, palinopsia, photophobia and nyctalopia. In the literature other symptoms included visual hallucinations altered motion perception, palinopsia, tracers and color enhancement. Ophthalmic and neurologic investigations were mostly normal. The majority of patients had ongoing symptoms. Two of our patients fully recovered—one after treatment with benzodiazepine and one without treatment. Twenty-five percent of cases from the literature fully recovered.

**Conclusions:**

HPPD presents with heterogeneous visual phenomena on a background of previous classic and non-classic hallucinogen use. Ophthalmic investigations are typically normal. The symptoms of HPPD in our case series overlap with the typical features of Visual Snow Syndrome (VSS). Patients presenting with VSS should be screened for past recreational drug use. The DSM-5 description of HPPD does not include visual snow, nyctalopia, photophobia or floaters. A revision of the diagnostic criteria to include these symptoms may better reflect the typical clinical phenotype. Increased awareness of HPPD as a secondary cause of VSS can avoid extensive investigations. Controlled trials comparing primary and secondary VSS patients are needed to understand the pathophysiology better and optimize treatment for HPPD.

## Introduction

Hallucinogenic substances are associated with a broad spectrum of visual hallucinations, distortions and illusionss (1, 2). Hallucinogen persisting perception disorder (HPPD) is characterized by re-emergence of symptoms experienced during acute hallucinogen intoxication following drug cessation (3). Symptoms may be intermittent or constant (4). The Diagnostic and Statistical Manual of Mental Disorders, 5th Edition (DSM-5) distinguishes HPPD from common post-drug use “flashbacks” by the syndrome's association with significant distress or functional impairment (3). A review by Lerner et al. proposed that recurrent perceptual disturbances post-substance use be classified into two syndromes—HPPD 1 referring to transient and benign re-experiencing of perceptual symptoms and HPPD 2 describing chronic and highly distressing visual disturbances (5). These criteria have not been accepted by DSM-5.

Hallucinogens can be broadly categorized as “classical” or “non-classical” hallucinogens (6) (Table 1). Classic hallucinogens are defined by agonist effects at 5-HT2A receptors, including lysergic acid diethylamide (LSD), psilocybin, mescaline, and N-dimethyltryptamine (DMT). The designer drugs “NBOMes” (N-benzylmethoxy derivatives) also work via this mechanism (7). Non-classical hallucinogens can be subdivided into entactogens such as 3,4-methylenedioxymethamphetamine (MDMA), dissociatives (N-methyl-D-aspartate receptor [NMDA] antagonists) including ketamine, phencyclidine and ibogaine and specific muscarinic antagonists like scopolamine (“devil's breath”) (6, 8). Cannabinoid agonists (i.e., tetrahydrocannabinol [THC]) have also been described to possess hallucinogenic properties (9) and are sometimes considered a subclass of hallucinogens (6). HPPD has traditionally been associated with classic hallucinogens (5) however entactogens, dissociatives, cannabinoids, and atypical hallucinogens have all now been implicated in the development of the disorder (10, 11).

**Table 1:** Classification of hallucinogens.

**Class**	**Pharmacologic mechanisms**	**Effects**	**Drugs**
Classic hallucinogens	5-HT2A agonists	Visual and auditory hallucinations	LSD, DMT, Psilocybin
Entactogens	Monoamine releasers and reuptake inhibitors	Evoke a sense of openness and connection	MDMA
Dissociatives	NMDA antagonists	Dissociative and anesthetic effects	Ketamine DXM Phencyclidine
Muscarinic receptor antagonists		Hallucinations with mood and cognitive disturbances	Scopolamine

HPPD is considered rare. Previous analyses estimated the disorder occurs in 1 of 50,000 hallucinogen users (12), however, in a recent web-based questionnaire by Baggott et al. 4.2% of 2,455 surveyed individuals described persistent visual phenomena post-drug use associated with clinically significant distress consistent with HPPD (13). True estimates are challenging due to a lack of large case series and population studies (14). The overlap of HPPD with drug-induced psychosis and other differential diagnoses such as migraine aura without headache, focal epilepsies, visual snow syndrome (VSS) and narcolepsy-cataplexy syndrome further complicates the epidemiology (13, 15, 16).

HPPD has previously been compared with VSS. VSS is another poorly understood condition characterized by the presence of persistent grainy, pixelated vision (“visual snow”), commonly accompanied by positive visual phenomena such as palinopsia, entopic phenomena, photophobia and nyctalopia. Unlike HPPD, VSS is defined by the absence of previous psychotropic substance use triggering the onset of symptoms. Typically VSS cannot be attributed to a clear provoking factor (17).

There are few HPPD case reports in the neurologic or ophthalmic literature and no trials investigating visual function. This paper aimed to provide a qualitative description of the visual symptoms, illicit drug use, outcomes, neuro-ophthalmic investigations and treatment of HPPD patients referred to tertiary neuro-ophthalmology outpatient clinics. The literature was reviewed for case reports to examine the commonly associated drugs, visual characteristics, investigation, treatments and recovery rates of HPPD patients. Additionally, we draw parallels between the features of HPPD and VSS.

## Materials and Methods

Fourteen HPPD cases were contributed in de-identified format from four neuro-ophthalmologists employed across five Australian health care sites. To be included, cases had to meet DSM-5 HPPD diagnostic criteria (3). While DSM-5 refers to the re-experiencing of perceptual symptoms following hallucinogen use, given the number of reports describing HPPD with other recreational drugs (5) we did not limit cases to patients who had only used typical hallucinogens. Due to diagnostic uncertainty, one patient was excluded, leaving 13 cases for review.

Data was collected on visual symptoms, illicit drug(s) used prior to symptom onset, visual examination findings, neurologic and ophthalmologic investigations, treatment and recovery. Twenty-four visual symptoms were specified based on DSM-5 HPPD diagnostic criteria (3) and a summary of common symptoms from a comprehensive systematic literature review by Martinotti et al. (2). Visual phenomena not captured by these references were listed as “unspecified.” MEDLINE and PubMed databases were searched for HPPD case reports published between 2000 and 2020 using the terms “hallucinogenic persisting perception disorder” OR “hallucinogen persisting perception disorder” and “case reports.” The search yielded 28 publications. Ten studies were excluded. One did not describe case reports, five were not related to HPPD, one had minimal clinical information, one was not available in English, and one case did not meet HPPD diagnostic criteria due to a lack of substance use. Twenty-five case reports across the remaining 18 publications were reviewed. One case was excluded as symptoms occurred only during acute intoxication, leaving 24 cases.

Descriptive statistical analyses were performed with SPSS version 24; IMB Corp., 2016. Continuous data, where available, is described as averages with standard deviations (SD).

## Results

### Background Features

Features of the 13 HPPD cases are displayed in Table 2. Our cohort includes 12 males and one female at a median age of 26 years.

**Table 2:** Characteristics of HPPD cases.

**Patient**	**Age**	**Gender**	**Drug(s) used closest to symptoms**	**Other drugs previously**	**Symptoms**
1	17	M	Methamphetamine		Visual snow, floaters, photopsia, tracers, nyctalopia
2	26	M	Natural THC		Visual snow, visual hallucinations, floaters, photophobia, photopsia, tracers
3	33	M	LSD		Visual snow, visual hallucinations, photopsia, palinopsia, geometric phosphenes
4	25	M	Psilocybin	Synthetic THC	Visual snow
5	36	M	MDMA, methamphetamine		Visual snow, altered motion perception, color enhancement, palinopsia, micropsia, halos, micropsia
6	26	M	LSD		Visual snow, photopsia, tracers, palinopsia, halos, floaters, photophobia, nyctalopia
7	25	F	LSD		Visual snow, palinopsia, floaters, photophobia
8	25	M	LSD	MDMA	Visual snow, photopsia, palinopsia, floaters, photophobia, nyctalopia
9	27	M	Synthetic + natural THC		Palinopsia, floaters, metamorphopsia, photophobia, nyctalopia, monocular vertical diplopia, enoptic phenomena
10	40	M	Methamphetamine, cocaine, MDMA		Visual snow, photopsia, palinopsia, floaters, photophobia, nyctalopia, enoptic phenomena
11	20	M	Uncertain of which drugs	Uncertain of which drugs used	Visual snow
12	34	M	MDMA	LSD, THC, cocaine, psilocybin, MDMA	Visual snow, altered motion perception, tracers, palinopsia, floaters, superimpositions, intense fragmentation, repetitions, geometric phosphenes, imagistic phosphenes, acquired dyslexia
13	22	M	25N-NBOMe	Ketamine, THC, cocaine, MDMA	Visual snow, photopsia, tracers, halos, floaters, keenness, repetitions, photophobia, nyctalopia

*LSD, lysergic acid diethylamide; MDMA, 3,4-Methyl enedioxy methamphetamine25N-NBOME (2-(2,5-dimethyoxy-4-nitrophenyl)-N-(2-methoxybenzyl)ethanamine); THC, Tetrahydrocannabinol*.

Illicit drugs used closest to HPPD symptom onset (suspected “trigger”) were recorded. Detailed information regarding the exact time between the suspected causative drug used and initial symptoms was not available. Drugs used >12 months prior to HPPD symptoms were listed separately. Six of 13 patients (46.2%) had used a classic hallucinogen (LSD, psilocybin, 25N-NBOMe) closest to the onset of symptoms. LSD was the most common suspected trigger overall (4 of 13). In three patients (23.1%), an entactogen (MDMA) had been consumed closest to HPPD onset. Two of these patients had combined stimulants (cocaine, methamphetamine) with MDMA. In two patients, cannabinoids were the suspected trigger for HPPD symptoms—one had used natural THC alone, and another a combination of synthetic and natural THC. One patient had used methamphetamine closest to onset of HPPD symptoms and denied any classic or non-classic hallucinogenic substance use at any stage. One patient was unsure of which illicit drugs he had previously consumed.

The frequency of visual symptoms are reported in Table 3. Twenty-two different symptoms were reported. We identified five features not typically associated with HPPD—nyctalopia, photophobia, entoptic phenomena, metamorphopsia and monocular vertical diplopia. All patients described visual snow. More than half experienced persistent floaters (61.5%), palinopsia (53.8%) and/or photopsia (53.8%). Photophobia was described by seven patients (53.8%). No patients reported macropsia (3), pareidolia, visualizations, fractals, recurrent synaesthesia, distorted perception of distance or monochromatic vision (2).

**Table 3:** Frequency of visual symptoms amongst HPPD cases (*n*, %).

**Symptoms specified by DSM-5 criteria**	
**Palinopsia**	**7 (53.8)**
**Photopsia**	**7 (53.8)**
**Tracers**	**5 (38.5)**
**Halos**	**3 (23.1)**
**Visual hallucinations**	**2 (15.4)**
**Altered motion perception**	**2 (12.4)**
**Color enhancement**	**1 (7.7)**
**Micropsia**	**1 (7.7)**
**Common symptoms not specified by DSM-5 criteria**	
**Aeropsia/visual snow**	**13 (100)**
**Floaters**	**8 (61.5)**
**Repetitions**	**2 (15.4)**
**Geometric phosphenes**	**2 (15.4)**
**Superimpositions**	**1 (7.7)**
**Intense fragmentation**	**1 (7.7)**
**Keenness**	**1 (7.7)**
**Imagistic phosphenes**	**1 (7.7)**
**Acquired dyslexia**	**1 (7.7)**
**Unspecified associated visual symptoms**
**Photophobia**	**7 (53.8)**
**Nyctalopia**	**6 (46.2)**
**Entoptic phenomena**	**2 (15.4)**
**Metamorphopsia**	**1 (7.7)**
**Monocular vertical diplopia**	**1 (7.7)**

### Examination and Investigations

Examination and investigation were normal in the majority of patients. Visual acuity (VA) was examined in 11 patients, and automated perimetric visual field assessment was available in 10. VA was normal in all patients, as were visual fields. Color vision was examined using the Ishihara test in 10 patients. Case 10 had significantly reduced color perception with 5/17 OS (oculus sinister, left eye) and 4/17 OD (oculus dexter, right eye). All other patients had normal color vision.

Nine patients had an MRI (magnetic resonance imaging) brain performed. Seven had no abnormalities. Two had minor abnormalities. Case 1 had a developmental change in the lingual gyrus, and case 8 had non-specific scattered T2-hyperintense lesions. Both were deemed incidental findings and clinically insignificant.

Optical coherence tomography (OCT) was performed in eight patients. Two had results listed as “normal,” and six had specific measurements recorded. All OCT findings fell within normal ranges (results available as a Appendix A1). Electroretinography and visual evoked potentials were performed in two patients and reported as “normal.” Detailed results were not available.

### Treatment and Recovery

Twelve patients had data available on treatment, and eight patients had recovery status recorded (Table 4). Two patients fully recovered, two partially recovered, and four did not recover.

**Table 4:** Treatment and recovery status of HPPD cases.

**Patient**	**Treatment**	**Recovery status**
1	Tinted lenses	No recovery
2	None	No recovery
3	SSRIs (4 years), then NRIs (ongoing)	Partial
4	BZDs	Full recovery
5	None	No recovery
6	Psychologic therapy + AEDs	Unknown
7	AEDs	Unknown
8	None	Unknown
9	None	No recovery
10	None	Unknown
11	None	Unknown
12	Psychologic therapy, AEDs (lamotrigine), BZD (lorazepam for 2 years, then clonazapem), SSRI (escitalopram) VSI online delivery of sensory intervention	Partial
13	None	Full after taking MDMA

*AED, Antiepileptic Drug; BZD, benzodiazepine; NRI, Norepinephrine Reuptake Inhibitors; SSRI, Selective Serotonin Reuptake Inhibitors; VSI, Visual Snow Intervention*.

Six out of 12 patients did not receive treatment, four of which had their recovery status recorded. Three did not recover, and one patient (case 13) fully recovered after acute MDMA intoxication. This patients' HPPD had been attributed to 25N-NBOMe on a background of prior MDMA, ketamine, cocaine and natural THC use.

Three patients were treated with anti-epileptic drugs (AED). Case 7 was treated with an AED alone, and case 6 received additional psychological therapy. The AEDs for these two patients were not specified, and their recovery status was unknown. Case 12 partially recovered after treatment with lamotrigine combined with benzodiazepines (lorazepam, followed by clonazepam), a selective serotonin reuptake inhibitor (SSRI) (escitalopram), psychological therapy and a visual snow sensory intervention involving online delivery of staring at static.

One patient fully recovered after treatment with benzodiazepines, and one patient partially recovered with an SSRI and serotonin-norepinephrine reuptake inhibitor (SNRI). The agents used were not specified. One patient was treated with tinted glasses and did not improve.

## Literature Review

Twenty-four HPPD case reports were identified. The features of these cases, including implicated drugs, symptoms, investigations, treatment and recovery, are synthesized in Table 5. The mean age of first drug use was 19.5 ± 3.7 years, and mean age of symptom onset was 24.3 ± 6.6 years. There was a male to female ratio of 2.4:1.

**Table 5:** Literature review of clinical features, investigations, management and outcome of HPPD case reports (*n* = 24).

**Author**	**Drug use**	**Symptoms**	**Investigations**	**Treatment**	**Recovery**
Hermle (18)	LSD, natural THC, MDMA, ketamine, psilocybin	Altered motion perception, palinopsia, halos, micropsia, macropsia, visualizations	MRI, median nerve SSEP, EEG: normal Neuropsychology: dysthymia	SSRI, AED, psychology	Partial
Kurtom (19)	LSD, natural THC	Visual hallucinations	Neuroimaging (unspecified): normal	BZD	Full
Lerner (20)	LSD, natural THC	Micropsia, macropsia, altered distance perception	EEG: normal	None	Full
Skyrabin (21)	MDMA	Color enhancement, tracers, palinopsia, floaters, visual snow	CTB, MRI: normal MRA/MRV: insignificant blood flow asymmetry EEG: mild abnormalities	SSRI, BZD, TCA, AED	Partial
Skyrabin (21)	LSD, natural + synthetic THC, MDMA, cocaine	Color enhancement, palinopsia, halos, floaters, pareidolia, visual snow	MRI, carotid/vertebral artery USS: both mild abnormalities	SSRI, BZD, AED	Partial
Brodrick (22)	LSD, natural THC, cocaine, PCP	Altered motion perception, color enhancement, micropsia, macropsia	MRI: mild abnormalities	SSRI, AED, TeCA	Suicide
Subramanian (23)	LSD, natural THC, MDMA, psilocybin	Visual hallucinations	CTB, EEG: normal	BZD, beta blocker, antipsychotic	Partial
Aldurra (24)	LSD	Visual hallucinations, floaters	None	SSRI, antipsychotics	Partial
Lerner (25)	LSD, natural THC, MDMA	Altered motion perception, photopsia, tracers, floaters	None	SSRIs, SNRI	Partial
Neven (26)	LSD, natural THC, cocaine, MDMA	Visual hallucinations, recurrent synaesthesia	MRI, EEG: normal	AEDs, SSRI, BZDs, antipsychotics, SARI	Suicide
Lerner (27)	LSD, natural THC, MDMA	Visual hallucinations, photopsia, tracers, palinopsia	None	BZDs, antipsychotics	Partial
Lerner (27)	LSD, natural THC	Visual hallucinations, tracers	None	BZDs, antipsychotics	Partial
Anderson (28)	Natural THC, MDMA, methamphetamine	Visual hallucinations, altered motion perception, palinopsia, color enhancement, halos, macropsia	MRI, neuropsychology: normal EEG: occipital lobe epilepsy	AED	Full
Lerner (11)	Natural + synthetic THC	Altered motion perception, color enhancement, tracers, palinopsia, halos, visual snow	EEG: normal	BZD	Partial
Lerner (11)	Natural + synthetic THC	Visual hallucinations, altered motion perception, tracers palinopsia, halos, floaters, visual snow	None	BZD	Partial
Gaillard (10)	Natural THC	Visual hallucinations, altered motion perception, palinopsia, visual snow	ERG: normal	None	Partial
Gaillard (10)	Natural THC	Altered motion + depth perception	MRI, EEG: normal	None	None
Gaillard (10)	LSD	Visual hallucinations, superimpositions	EEG: toxic encephalopathy	AED	Partial
Sunness (29)	LSD, natural THC	Palinopsia	Fluorescein angiography, ERG: normal	None	Unknown
Iaria (30)	LSD, natural THC, cocaine, psylocybin	Pareidolia	MRI: normal fMRI: hallucinations associated with increased and decreased neural activity in cortical/subcortical regions Neuropsychology: normal	AEDs, antipsychotics	Unknown
Lerner (31)	LSD, natural THC, cocaine, MDMA	Altered motion perception, color enhancement	EEG: normal	None	Full
Lerner (11)	LSD, natural THC, MDMA	Visual hallucinations, tracers	None	None	Partial
Knuijver (32)	LSD, natural THC, ibogaine, methamphetamine	Visual hallucinations	None	EMDR	Full
Coppola (33)	Psilocybin, natural and synthetic THC	Visual hallucinations, altered motion perception, tracers, palinopsia, halos, geometric phosphenes	Neuropsychology: normal	BZD	Full

*AED, Antiepileptic Drug; CTB, Computed Tomography Brain; BZD, benzodiazepine; EEG, electroencephalogram; EMDR, Eye Movement Desensitization and Reprocessing; fMRI, Functional Magnetic Resonance Imaging; LSD, Lysergic Acid Diethylamide; MDMA, 3,4-Methylenedioxymethamphetamine; MRA, Magnetic Resonance Angiogram; MRI, Magnetic Resonance Imaging; MRV, Magnetic Resonance Venogram; SARI, Serotonin Antagonist and Reuptake Inhibitors; SSEP, somatosensory evoked potential; SSRI, Selective Serotonin Reuptake Inhibitors; SNRI, Serotonin and Norepinephrine Reuptake Inhibitors; TeCA, Tetracyclic Antidepressant; THC, Tetrahydrocannabinol; USS, Ultrasound*.

Nine different drugs were associated with HPPD—LSD (17), psilocybin (3), ketamine (1), PCP (1), MDMA (10), methamphetamine (2), cocaine (5), ibogaine (1) and THC [natural (21) and synthetic (3)]. All cases were associated with a classic or non-classic hallucinogen(s). Six patients (25%) had never used any classic hallucinogenic substance. LSD was the most frequent classic hallucinogen reported [*n* = 17/24 [70.8%]], and THC was the most reported drug overall [*n* = 21/24 [87.5%]] however was used by most patients in association with LSD or psilocybin. Four patients developed HPPD after isolated natural and/or synthetic THC intoxication, and one patient used THC with MDMA and methamphetamine before symptom onset.

Seventeen different symptoms were described. Visual hallucinations were the most common symptom amongst literature case reports [*n* = 13/24 [54.2%]] compared with no formed visual hallucinations reported by our cases. Five case reports described visual snow (20.8%) compared with 100% of our case studies. Other symptoms in the literature included altered motion perception (10), palinopsia (10), tracers (trails) (8), color enhancement (6), halos (6), floaters (5), macropsia (4), micropsia (3), pareidolia (2), distorted perception of distance (2), photopsia (2), visualizations (1), superimpositions (1), recurrent synaesthesia (1) and geometric phosphenes (1).

An EEG was performed in 41.7% of case reports. The majority were normal. One patient had an initial EEG demonstrating bilateral occipitotemporal epileptiform discharges; however these could not be confirmed in later EEGs. Neuroimaging was completed in 10 patients, most commonly MRI. All were reported as normal or with non-specific abnormalities. Other investigations included electroretinograms (2), median nerve somatosensory evoked potentials (1) and neuropsychological testing (2).

Most cases received treatment. The most common therapies were benzodiazepines [*n* = 10/24 [41.7%]], AEDs [*n* = 8/24 [33.3%]], SSRIs [*n* = 7/24 [29.2%]] and antipsychotics [*n* = 6/24 [25%]]. Other treatments included SNRIs, tetracyclic antidepressants (TeCAs), tricyclic antidepressants (TCAs), serotonin antagonist and reuptake inhibitors (SARIs), beta blockers, psychological therapy and eye movement desensitization and reprocessing (EMDR) (*n* = 1 for all the aforementioned).

Twenty-five percent of patients fully recovered, and 54.2% partially recovered. Three patients had no symptom recovery, two of which committed suicide. The remaining two patients did not have their recovery status recorded.

## Discussion

We describe 13 cases of HPPD with the inclusion of visual assessments demonstrating that visual acuity, visual fields and OCT are typically normal in this patient group. This highlights the importance of a careful history in the assessment of patients presenting with positive visual phenomena, focusing on timeline and relationship to substances and medications. In the absence of other neurologic features, a diagnosis of HPPD can be elicited from history alone without unnecessary specialist investigations.

All of our cases described visual snow. The other most common symptoms experienced by patients were floaters, palinopsia, photophobia, photopsia and nyctalopia. Currently, the DSM-5 does not include visual snow, nyctalopia, photophobia or floaters in their description of HPPD. A revision of the diagnostic criteria to include these symptoms may better reflect the common presentations and guide clinicians in identifying patients presenting with a suggestive history.

The clinical phenotype of HPPD presented in our paper shares overlapping features with visual snow syndrome (VSS) (Table 6). This was previously described in a large case series by Puledda et al. (17). VSS and HPPD patients in this series experienced a similar number of visual symptoms, and the most frequently reported for both were visual snow, floaters, palinopsia, photophobia and nyctalopia. These symptoms are comparable with the most commonly reported by our cases, and in the literature, one in five case reports experienced visual snow with HPPD. Both VSS and HPPD lack objective ophthalmic findings (3, 34) and the major factor differentiating the two syndromes is the use of recreational drugs within the year prior to symptom onset (17). HPPD also tends to occur with a more abrupt onset and at a later age compared with VSS (17). The development of chronic visual disturbances is common to both HPPD and VSS. However, this may reflect a lack of evidence-based treatments for both conditions (35). The similar presentations and lack of supportive investigations HPPD and VSS again emphasized the need for detailed history-taking in patients with unexplained visual symptoms. The two syndromes should be differentiated so that patients with HPPD can be appropriately counseled on avoiding potential triggering substances.

**Table 6:** Comparison of HPPD and VSS clinical features.

	**HPPD**	**VSS**
Age of onset	Early adulthood	Late childhood
Gender predominance	Male	Genders equally affected
Common symptoms	Visual snow, floaters, palinopsia, photophobia, photopsia	Visual snow, floaters, palinopsia, photophobia, nyctalopia
Presentation	Abrupt	Variable
Triggers	Recreational substances	Unknown
Treatments	Benzodiazepines, AEDs	Benzodiazepines

The pathophysiology of HPPD remains poorly understood. While LSD was the most common hallucinogen associated with symptom onset, in over half our cases and one in four literature reports classic hallucinogens did not trigger HPPD. Cannabinoids (synthetic and natural) and MDMA were common; two of our patients and five from the literature experienced the onset of symptoms after taking cannabinoids, and three of our patients and two from the literature developed HPPD after consuming MDMA without associated consumption of a classic hallucinogen. HPPD secondary to dissociatives, entactogens and cannabinoids has previously been described (2, 5). The range of associated psychotropics may indicate the involvement of multiple mechanisms in the pathophysiology. Further research is required to understand the neuropharmacology of the disorder (5). Currently, the DSM-5 defines HPPD as occurring in the context of previous hallucinogen intoxication however the diagnosis should be considered in patients with suggestive clinical features and a history of recreational drug use beyond the classic hallucinogens.

The management of HPPD is based on case studies due to a lack of controlled trials (2, 14). Our cohort treatment included a range of pharmacologic and non-pharmacologic measures, and management amongst the literature was similarly varied. Benzodiazepines and AEDs were the most frequently prescribed treatments. Full recovery is uncommon and occurred in only two of our patients, and 25% of literature cases reported. One of our patients and two literature case reports fully recovered without treatment, indicating a subset of patients may experience self-limited symptoms. Benzodiazepines were the only pharmacologic intervention associated with complete recovery in our cases and those from the literature. AEDs without co-prescription of benzodiazepines resulted in complete recovery in one literature case study but not in our patients. Previous reviews have demonstrated benzodiazepine to effectively alleviate HPPD symptoms (2, 5), and in a recent web-based prospective questionnaire of treatment responses in VSS by Puledda et al. benzodiazepines provided modest symptomatic benefit (36). AEDs have shown some efficacy for treatment of HPPD (2, 5); however responses in VSS vary. A review of VSS treatments by Van Dongen et al. lamotrigine provided symptomatic improvement in a minority of patients (35), whereas in the questionnaire by Puledda et al. AEDs (including lamotrigine) were more likely to worsen visual symptoms (36). The overall evidence for treatment of both disorders is scarce (2, 37). The potential benefits of AEDs in HPPD compared with ineffectiveness or possible harm in VSS requires further investigation.

Our study has several limitations. Neuro-ophthalmologists involved in this study all research VSS. This may have biased the frequency of visual snow as patients with HPPD experiencing this symptom were more likely to be referred. Patient numbers for both our cases and literature reports were small. The study was observational. Therefore, variables such as age and gender were not controlled. Cases were collected retrospectively, meaning data for treatment outcomes were not available for all patients. All patients had their drug history recorded; however detailed timelines between use of illicit substance and onset of HPPD symptoms was not available. Three patients could not recall all drugs used. The type of investigations performed varied, and two patients had no neuroimaging or ophthalmic testing performed.

In conclusion, HPPD is characterized by positive visual phenomena with no clear supporting investigations or examination findings. A careful history combined with an awareness of the commonly associated symptoms can elicit the diagnosis and avoid extensive investigations. Differentiating HPPD from classical VSS is important for appropriate treatment. Future studies comparing VSS and HPPD may be useful in examining whether they are two distinct conditions with shared pathophysiologic mechanisms or different aspects of the same disorder. Randomized controlled trials are required to better understand the neurobiological mechanisms and optimal treatments for HPPD.

## Data Availability Statement

The original contributions presented in the study are included in the article/supplementary material, further inquiries can be directed to the corresponding author/s.

## Ethics Statement

Written informed consent was obtained from the individual(s) for the publication of any potentially identifiable images or data included in this article.

## Author Contributions

All authors listed have made a substantial, direct, and intellectual contribution to the work and approved it for publication.

## Conflict of Interest

The authors declare that the research was conducted in the absence of any commercial or financial relationships that could be construed as a potential conflict of interest.

## Publisher's Note

All claims expressed in this article are solely those of the authors and do not necessarily represent those of their affiliated organizations, or those of the publisher, the editors and the reviewers. Any product that may be evaluated in this article, or claim that may be made by its manufacturer, is not guaranteed or endorsed by the publisher.

## Appendix

**Appendix 1:** Results of optical coherence tomography.

**Patients, *n***	**8**	
	**Results (mean** **±SD)**, **μm**	**Reference range**
L) RNFL (*n* = 6)	95.17 (9.5)	92.8 (9.4)
L) macular (*n* = 5)	268 (7.1)	262.8 (13.3)
L) GCLIP (*n* = 4)	83 (5.5)	82.1 (6.2)
R) RNFL (*n* = 6)	96.5 (9.0)	92.8 (9.4)
R) macular (*n* = 5)	269 (4.8)	262.8 (13.3)
R) GCLIP t (*n* = 4)	84 (6.0)	82.1 (6.2)

*GCLIP, Ganglion Cell Layer Inner Plexiform; RNFL, Retinal Nerve Fiber Layer; L), Left; R), Right*.
